# Chronic *Candida albicans* meningoencephalitis in a patient with mantle cell lymphoma: a diagnostic challenge

**DOI:** 10.1186/s42466-025-00375-2

**Published:** 2025-03-03

**Authors:** Johannes L. Busch, Justus Schikora, Lisa-Marie Wackernagel, Jawed Nawabi, Matthias Endres, Klemens Ruprecht

**Affiliations:** 1 Department of Neurology, Charité - Universitätsmedizin Berlin, Berlin, Germany; 2 Berlin Institute of Health at Charité– Universitätsmedizin Berlin, BIH Biomedical Innovation Academy, BIH Charité Junior Clinician Scientist Program, Berlin, Germany; 3 Department of Infectious Diseases, Respiratory Medicine Berlin and Intensive Care, Charité - Universitätsmedizin Berlin, Berlin, Germany; 4 Institute for Neuroradiology, Charité - Universitätsmedizin Berlin, Berlin, Germany; 5 Center for Stroke Research Berlin, Charité - Universitätsmedizin Berlin, Berlin, Germany; 6 ExcellenceCluster NeuroCure, Charité - Universitätsmedizin Berlin, Berlin, Germany; 7 German Center for Neurodegenerative Diseases (DZNE) Partner site Berlin, Berlin, Germany; 8 German Centre for Cardiovascular Research (DZHK), Partner site Berlin, Berlin, Germany; 9 German Center for Mental Health (DZPG), Partner site Berlin, Berlin, Germany

**Keywords:** Candida albicans, Meningitis, Meningoencephalitis, Encephalitis, Neurocandidiasis, Cerebrospinal fluid

## Abstract

Due to its unspecific clinical presentation and the multitude of possible etiologies, chronic meningoencephalitis in immunosuppressed patients often represents a diagnostic challenge. Here, we report the clinical, radiological, cerebrospinal fluid, and microbiological findings of a 54-year-old male immunocompromised patient with mantle cell lymphoma and a 2-month history of brainstem and spinal meningoencephalitis. After unsuccessful treatment trials with antibiotics, a *Candida albicans* infection was confirmed by biopsy of a spinal cord lesion and large-volume cerebrospinal fluid culture. Therapy with liposomal amphotericin B/flucytosine and subsequent fluconazole resulted in significant clinical improvement. This case illustrates the importance of identifying the underlying cause of chronic meningoencephalitides in immunocompromised patients.

## Main text

A 54-year-old male patient was admitted to the Department of Neurology, Charité - Universitätsmedizin Berlin due to a deterioration in his general condition. He was suffering from exertional dyspnea, had lost seven kilograms and complained of headaches, nausea, and vomiting. His symptoms had developed insidiously over a period of about two months. The patient had been diagnosed with mantle cell lymphoma (Ann Arbor Stage IVb) one year earlier, for which he received six cycles of alternating R-CHOP/DHAP chemotherapy followed by autologous stem cell transplantation 6 months prior to admission. Staging after chemotherapy showed regression of lymphoma manifestations. Subsequently, two courses of rituximab maintenance therapy were administered 4 and 2 months prior to admission. Shortly after the second course of rituximab, the symptoms leading to the present admission started to develop.

On admission, the patient was afebrile and the neurological examination was unremarkable, except for subtle bilateral postural and action tremor. Laboratory findings included lymphopenia (0.45 /nl, reference 1.5–7.7 /nl), reduced serum IgG levels (5.6 g/l, reference 7.0–16.0 g/l) and normal C-reactive protein and lactate dehydrogenase levels.

**Fig. 1:**
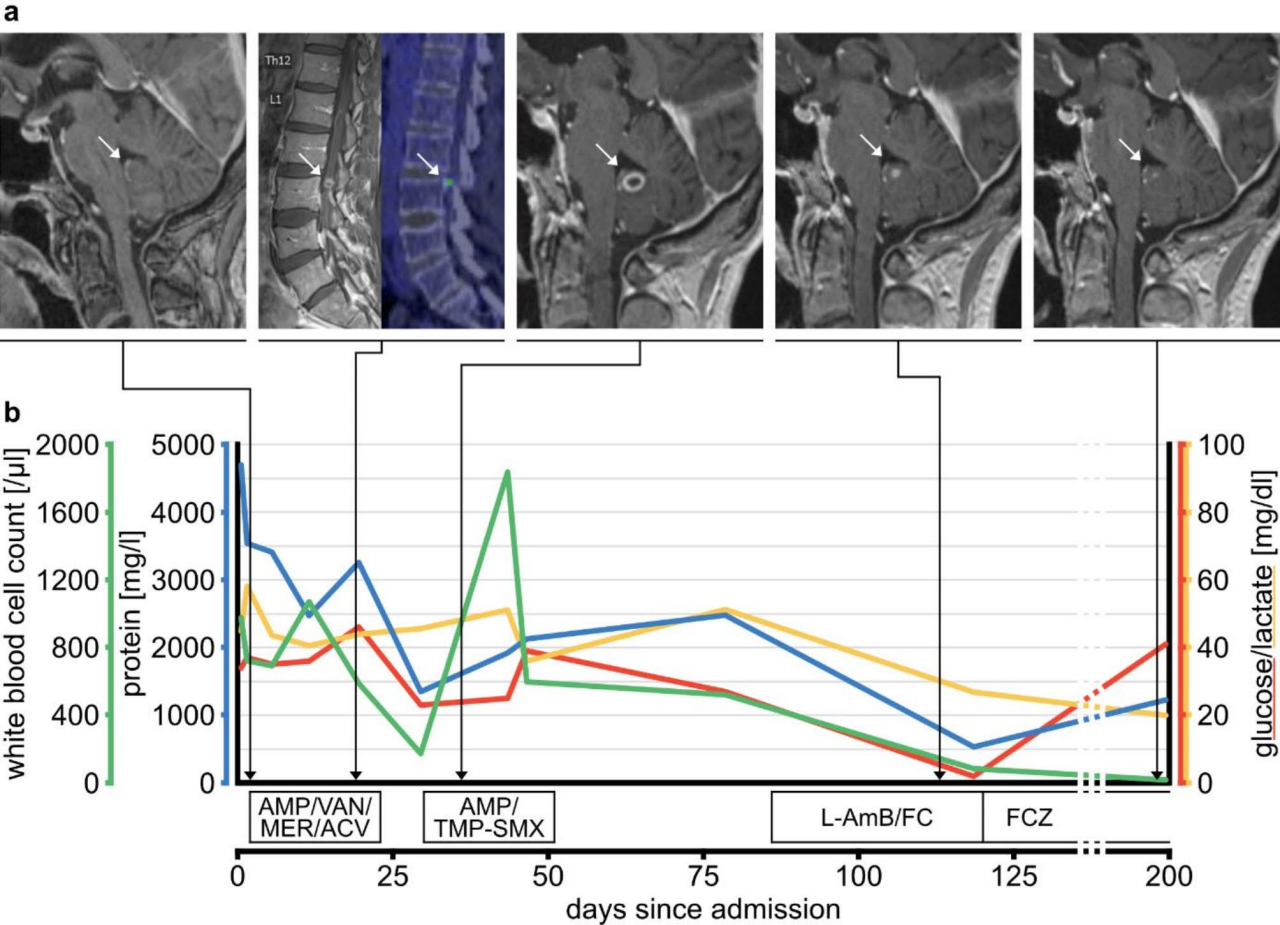
Radiological and CSF findings. (**a**) T1-contrast-enhanced MRI (day 2) showing contrast enhancement extending into the fourth ventricle via the left lateral aperture. T1-contrast-enhanced spinal MRI (day 16) and fluorodeoxyglucose positron emission tomography (day 19) demonstrating a spinal metabolically active lesion on the level of L2/3. During empirical antibiotic treatment, a ring-enhancing lesion formed on the floor of the fourth ventricle with cerebellar parenchymatous involvement (day 36). Antifungal therapy led to significant regression of the lesion. Lesions are highlighted by white arrows, respectively. (**b**) Longitudinal course of CSF cell count, glucose, lactate, and protein. AMP = ampicillin, VAN = vancomycin, MER = meropenem, ACV = acyclovir, TMP-SMX = trimethoprim/sulfamethoxazole, L-AmB = liposomal amphotericin B, FC = flucytosine, FCZ = fluconazole

Cerebral magnetic resonance imaging (MRI) revealed a left perimedullary signal inhomogeneity with pronounced peripheral contrast enhancement extending via the left foramen of Luschka cranially along the floor of the fourth ventricle (Fig. 1a). Additionally, adjacent intraparenchymal and circular peripheral contrast enhancement was observed in the right paravermal area. These findings were not visible in a prior cerebral MRI conducted 43 days before admission. Computed tomography imaging of the neck, chest, abdomen, and pelvis revealed no evidence of lymphoma recurrence.

Cerebrospinal fluid (CSF) analysis showed a mixed lymphocytic-granulocytic pleocytosis with 976 cells/µl (reference < 5 cells/µl), elevated lactate levels (45 mg/dl, reference 10–22 mg/dl) and hypoglycorrhachia (34 mg/dl, reference 40–70 mg/dl), but no intrathecal immunoglobulin syntheses. Repeated microscopic examinations of CSF cells, CSF flow cytometry, as well as nanopore sequencing of cell free DNA from CSF [1] did not provide evidence for malignancy.

Empirical antimicrobial therapy with vancomycin, meropenem, ampicillin, and aciclovir was initiated and then continued for three weeks. Repeated bacterial, mycobacterial and fungal cultures, and next-generation sequencing of blood and CSF samples for bacterial, viral and fungal agents returned negative. On day 16, a follow-up brain MRI and a spinal MRI revealed progressive peripontine contrast enhancement and a lumbar ring-enhancing lesion, respectively. Fluorodeoxyglucose positron emission tomography (on day 19) demonstrated increased metabolic activity in the lesions detected on cerebral and spinal MRI. Likewise, follow-up CSF analyses were not suggestive of a response to the empirical antimicrobial therapy (Fig. 1b). Clinically, the patient remained weak, afebrile and did not develop new neurological deficits. Repeat cerebral MRI on day 28 showed progression of the peripontine lesion with ring-enhancement. Given the pronounced brainstem affection, a three-week treatment trial of ampicillin and trimethoprim/sulfamethoxazole was started for suspected listeriosis. However, this was not associated with clinical improvement and the patient continued to have fluctuating CSF pleocytosis as well as progression of the brainstem lesion on cerebral MRI on day 36.

Extensive evaluations for infectious etiologies revealed borderline elevated levels of beta-D-glucan in serum and substantially elevated levels of beta-D-glucan in CSF (901 pg/ml, reference < 200 pg/ml [2, 3]). While further diagnostic investigations had to be delayed because of a SARS-CoV-2 infection, on day 65, a biopsy of the lumbar abscess-like formation and consecutive next-generation sequencing demonstrated the presence of *Candida* spp. DNA in the tissue sample. Subsequently, a repeat fungal culture, using a sample volume of 15 ml CSF, was positive for *Candida albicans* without drug resistance. Further assessments, including eye examination, a full body CT scan and transesophagal echocardiography, did not provide evidence for systemic candidiasis. Altogether, based on the detection of Candida albicans in the biopsy of the lumbar abscess-like formation and in CSF by next-generation sequencing as well as in a CSF culture, and absence of peripheral Candida manifestations, a diagnosis of chronic isolated neurocandidiasis was made.

Antifungal therapy with liposomal amphotericin B (300 mg/day) and flucytosine (6000 mg/day) was initiated on day 86, leading to significant clinical and radiological improvement and a decrease in the CSF cell count. Due to side effects of flucytosine (pancytopenia and acute cholecystitis), antifungal therapy was de-escalated to oral fluconazole (400 mg/day) on day 120. The patient tolerated this therapy well and was discharged on day 140 with continued intake of oral fluconazole. At a follow-up examination 2.5 months after discharge, the patient had recovered almost completely, only complaining of mild weakness in the legs. MRI showed further regression of all lesions and CSF analysis revealed a markedly reduced pleocytosis (16 cells/µl, Fig. 1b).

Candidemia accounts for 3% of bloodstream infections [4], and autopsy reports indicate that *Candida spp.* infection of the central nervous system (CNS) may develop in up to 50% of patients with systemic candidiasis [5]. Neurocandidiais may manifest as (sub)acute [6] or chronic meningitis/meningoencephalitis [7], micro- [5] and/or macroabscesses [8], which can also occur in the absence of systemic manifestations, as observed in our patient. Clinically, chronic neurocandidiasis presents with unspecific, slowly progressive symptoms. In a series of 15 adults and 3 neonates, headache was present in 87% of adults and fever in 83% of all patients, while neurologic signs, such as cranial nerve palsies (33%), confusion (33% of adults), and cognitive deterioration (27% of adults), were less frequent [7]. 

CNS candidiasis is primarily caused by *Candida albicans* and is mainly observed in (preterm) neonates, patients with a history of neurosurgery, and in immunocompromised patients [8]. As neutrophils are integral for the immune response to *Candida* spp [9], chemotherapy-induced neutropenia may have contributed to a primary blood-stream infection leading to secondary CNS invasion in our patient. Furthermore, our patient had been extensively treated with antibiotics and corticosteroids, which represent additional risk factors [10]. Still, in a prior series of 18 patients with chronic neurocandidiasis, no risk factor could be identified in 5 patients (28%) [7]. 

Diagnostic recommendations for suspected neurocandidiasis include fungal cultures using large volumes of CSF (15–20 ml), evaluation for systemic candidiasis (blood culture, funduscopy, biopsy of skin lesions) [8], and assessment of biomarkers of fungal infection (beta-D-glucan, Candida mannan antigen and antibodies) [11, 12]. However, as elevated levels of CSF beta-D-glucan have so far only been reported in case series, its diagnostic value has not yet been fully determined [13]. More recent guidelines for patients with hematological malignancies and putative CNS infections emphasize the role of early biopsy if focal lesions are present [14]. Indeed, also in our patient biopsy was key to the identification of neurocandidiasis and the CSF cultures became positive for *Candida albicans* only when using a large volume of CSF.

Treatment recommendations for neurocandidiasis include initial intravenous liposomal amphotericin B and flucytosine followed by oral fluconazole [14]. While this was associated with marked improvement in our patient, even in adequately treated patients mortality of chronic neurocandidiasis may be as high as 33% [7]. Standard infection control measures are crucial for preventing invasive candidiasis while prophylactic antifungal treatment is reserved for selected high-risk patient groups [15]. 

Demographic change and the increased use of immunosuppressive therapies has led to growing numbers of invasive fungal infections [16]. This case therefore highlights the importance of considering fungal infections in the differential diagnosis of chronic meningoencephalitides in immunocompromised patients and underscores the role of CNS biopsies in making a diagnosis of neurocandidiasis.

## Data Availability

Not applicable.

## Abbreviations

CSF: Cerebrospinal fluid
MRI: Magnetic resonance imaging
CNS: Central nervous system
R-CHOP/DHAP: Rituximab/cyclophosphamide/doxorubicin/vincristine/prednisone and dexamethasone/high-dose cytarabine/cisplatin
